# Comparison of the Relative Abuse Liability of Electronic Cigarette Aerosol Extracts and Nicotine Alone in Adolescent Rats: A Behavioral Economic Analysis

**DOI:** 10.3390/ijerph17030860

**Published:** 2020-01-30

**Authors:** Andrew C. Harris, John R. Smethells, Mary Palumbo, Maciej Goniewicz, Mark G. LeSage

**Affiliations:** 1 Department of Medicine, Hennepin Healthcare Research Institute, Minneapolis, MN 55415, USA; jsmethells@gmail.com (J.R.S.); lesag002@umn.edu (M.G.L.); 2 Department of Medicine, University of Minnesota, Minneapolis, MN 55455, USA; 3 Department of Psychology, University of Minnesota, Minneapolis, MN 55455, USA; 4 Department of Health Behavior, Roswell Park Comprehensive Cancer Center, Buffalo, NY 14203, USA; Mary.Palumbo@RoswellPark.org (M.P.); maciej.goniewicz@roswellpark.org (M.G.)

**Keywords:** electronic cigarettes, nicotine, abuse liability, behavioral economics, drug self-administration, rats

## Abstract

*Background:* Characterizing the determinants of the abuse liability of electronic cigarettes (ECs) in adolescents is needed to inform product regulation by the United States Food and Drug Administration (FDA). We recently reported that Vuse Menthol EC aerosol extract containing nicotine and a range of non-nicotine constituents (e.g., menthol, propylene glycol) had reduced aversive effects compared to nicotine alone in adolescent rats, whereas Aroma E-Juice EC aerosol extract did not. The current study used a behavioral economic approach to compare the relative abuse liability of these EC extracts and nicotine alone in an i.v. self-administration (SA) model in adolescents. *Methods:* Adolescents were tested for the SA of EC extracts prepared using an ethanol (ETOH) solvent or nicotine and saline, with and without 4% ETOH (i.e., the same concentration in the EC extracts) in 23 h/day sessions. *Results.* Although acquisition of SA was faster for nicotine + ETOH compared to all other formulations, the elasticity of demand for all nicotine-containing formulations was similar. *Conclusions:* EC aerosol extracts did not have greater abuse liability than nicotine alone in adolescents. These data suggest that nicotine may be the primary determinant of the abuse liability of these ECs in youth, at least in terms of the primary reinforcing effects of ECs mediated within the central nervous system.

## 1. Introduction

The use of electronic cigarettes (ECs) has increased greatly among adolescents in recent years, and ECs are now the most popular tobacco product in youth [1,2,3,4]. The Food and Drug Administration Center for Tobacco Products (FDA CTP) has extended its regulatory authority over tobacco products to ECs, and has also recently declared adolescent EC use to be an epidemic [5,6]. Evaluating determinants of the adverse consequences of ECs (e.g., abuse liability) in adolescents is essential for informing FDA regulation of these products, and for managing the impact of ECs on public health. 

Animal models provide a critical tool for informing the FDA regulation of tobacco products, including ECs [7,8]. To this end, our lab has evaluated the effects of several EC liquids that contain nicotine and a range of EC constituents in preclinical addiction models [9,10,11,12]. This approach allows evaluation of the aggregate effects of these constituents on EC abuse liability. This is important for understanding the abuse liability of ECs, because certain non-nicotine constituents in ECs (e.g., menthol, minor alkaloids) can enhance the addiction-related effects of nicotine, or can be behaviorally active themselves [2,4,13,14]. Because these models involve parenteral (e.g., i.v., s.c.) administration rather than inhalational exposure, they allow for the study of the addiction-related effects of EC constituents within the central nervous system (CNS), largely independent of their peripheral (e.g., sensory) effects. Parenteral administration also allows i.v. self-administration (SA), which is often considered the “gold standard” for the modeling of addiction in animals because it involves response-contingent drug exposure as occurs in humans, whereas no inhalational models of EC aerosol SA have yet been established.

We previously found attenuated aversive/anhedonic effects of three different EC liquids compared to nicotine alone at a high dose of nicotine in an intracranial self-stimulation (ICSS) model [10,12]. The primary EC constituent propylene glycol (PG) also attenuated nicotine’s acute ICSS threshold-elevating effects at PG concentrations similar to those in EC liquid doses used in our previous studies [15]. Given that nicotine’s aversive effects can limit tobacco product use (e.g., [16,17,18,19,20]), an attenuation of nicotine aversion by non-nicotine constituents in ECs could potentially promote EC abuse liability. However, two of the EC liquids that produced attenuated ICSS threshold-elevating effects in our earlier studies did not differ from nicotine alone in an i.v. SA model in adult rats [9,12].

A limitation of our previous studies is that the EC liquids did not include constituents that result from heating and aerosolizing these liquids as occurs during EC use in humans. The relevance of these studies to EC use in adolescents may also be limited by our use of only adult rats, which can differ from adolescents in terms of their sensitivity to the addiction-related effects of nicotine and non-nicotine constituents [21,22,23]. To address these limitations, we recently compared the aversive effects of extracts prepared from EC aerosol and nicotine alone in adolescent rats [24]. These extracts should contain most, if not all, of the constituents in an EC aerosol, thereby providing more clinically relevant exposure conditions than EC liquids. Aversion was measured using conditioned taste aversion (CTA) rather than intracranial self-stimulation (ICSS), because CTA involves a shorter training protocol that can be readily conducted during the brief adolescent period. We found that CTA to the Vuse Menthol EC extract, but not Aroma E-Juice EC extract, was attenuated compared to nicotine alone [24]. The Vuse Menthol data are consistent with our previous ICSS findings using EC liquids in adult rats, and support further evaluation of the effects of these EC aerosol extracts in adolescents in preclinical addiction models. 

The goal of the current study was to compare the effects of Vuse Menthol EC extract, Aroma E-Juice EC extract, and nicotine alone, in a model of i.v. SA in adolescent rats. Recent data indicate a greater SA for EC aerosol extract compared to nicotine alone in adult rats under certain conditions [25], indicating that SA models are sufficiently sensitive for evaluating the abuse liability of EC aerosol extracts (see below for further discussion). In contrast to that study, the current study used a 23 h/day access SA model. This access schedule more accurately simulates nicotine pharmacokinetics in tobacco users, and can provide greater sensitivity to the effects of non-nicotine constituents than SA models involving shorter access durations (e.g., 2 h/day) [26,27,28]. Formulations in the present study were compared in terms of acquisition, maintenance and elasticity of demand, determined using a behavioral economic approach. Behavioral economics is a conceptual, methodological and analytical framework, involving the application of microeconomic concepts to study factors that control the consumption of drugs and other reinforcers [29]. Central to this approach is the concept of a demand curve, or the function describing the consumption of a reinforcer (e.g., drug) (*y*-axis) versus its unit price (*x*-axis). In drug SA models, the unit price is operationalized as the cost–benefit ratio of response requirement divided by the unit dose of drug. The slope of the demand curve provides an index of the “elasticity” of demand (i.e., the rate at which consumption decreases as the unit price increases), with lower elasticity of demand indicating greater reinforcing efficacy [29,30,31,32]. Demand has served as a sensitive, quantitative measure of the reinforcing efficacy of nicotine and other drugs in both humans and animals [33,34,35], including our prior studies evaluating the effects of EC liquids [9,12], and was therefore used as our primary outcome. 

## 2. Materials and Methods

### 2.1. Subjects

Male and female adolescent Holtzman Sprague–Dawley rats (Envigo, Indianapolis, IN, USA), aged 22–24 days old at arrival, were used. Rats were initially housed in a colony room with ad libitum access to food and water under a reversed 12-h light/dark cycle (lights off at 10:00 h) for 5 days prior to surgery, and during a 4- to 6-day postoperative recovery period. Food restriction started following the post-operative recovery period. Rats were initially given 13 g/day for one week, 16 g/day the following week, and then 18 g/day for the remainder of the protocol. Pilot studies indicated that this feeding regimen provided a level of restriction commonly used in adults, while accommodating the increased caloric needs during adolescent development. After recovery from surgery, these rats were housed in operant conditioning chambers for the remainder of the study.

### 2.2. Drugs and EC Liquids/Aerosols

To prepare nicotine alone, (−)-nicotine obtained from Sigma Chemical Co. (St. Louis, MO) was dissolved in sterile saline. Electronic cigarette (EC) aerosol extract was prepared using Aroma E-Juice Whole Tobacco Alkaloid (WTA), EC refill liquid (Dark Honey Tobacco flavor) (http://www.aromaejuice.com, Scottsdale, AZ, USA), or Vuse Menthol EC liquid (http://vusevapor.com, Winston-Salem, NC, USA). We chose the Aroma E-Juice product, because it is advertised as containing higher levels of minor alkaloids than other EC liquids, and produced less aversive/anhedonic effects compared to nicotine alone in an intracranial self-stimulation (ICSS) model in adults [12]. Based on the label, this EC liquid contained 80% vegetable glycerine (VG) and 20% propylene glycol (PG), and had a nicotine concentration of 24 mg/mL. The Vuse Menthol EC was studied because it is a popular EC [36], and because menthol can have addiction-related CNS effects and alter the pharmacokinetics and behavioral effects of nicotine [13,37,38,39]. We also found that Vuse Menthol EC extract produced reduced aversive effects compared to nicotine alone in adolescents in a conditioned taste aversion (CTA) model [24]. According to the manufacturer, Vuse Menthol EC liquid contained 48 mg/mL nicotine and an unspecified amount of menthol flavoring. No other ingredient information was provided for this product.

EC aerosol extracts were prepared as described in our prior study [24]. These extracts were derived from EC aerosol produced by a smoking machine under puffing conditions based on those in EC users [40]. Levels of nicotine and other EC constituents (e.g., minor alkaloids, PG, VG) in these extracts are reported in our prior study [24]. Following determination of their nicotine concentration, extracts were diluted to the appropriate nicotine concentration using saline. We used ethanol (ETOH) as a solvent because it allows the extraction of both volatile and non-volatile constituents that are water-soluble and non-water soluble. ETOH has also been used in numerous preclinical studies evaluating addiction-related effects of extracts prepared from cigarette smoke [41,42,43]. EC extracts contained 4% ETOH after dilution to the 0.06 mg/kg nicotine unit dose used for self-administration (SA). Based on the low volumes administered (≤0.03 mL), these concentrations resulted in ETOH doses ≤ 3.2 mg/kg ETOH for each nicotine infusion. To control for any effects of ETOH itself on NSA, nicotine alone and saline control solutions were used with and without ETOH at the same concentration as in the EC extracts (i.e., 4%). All formulations were sterile-filtered, and their pH was adjusted to 7.4 prior to use. Nicotine concentrations in solutions of nicotine alone and extracts were measured by gas chromatography, as described previously [44].

### 2.3. Surgery

Each rat was implanted with a chronic catheter into the right jugular vein under i.m. ketamine (75–90 mg/kg)/dexmedetomidine (0.25 mg/kg) anesthesia, as we have described previously [28,45]. Animals were allowed at least four days to recover from surgery. During this time, they received daily i.v. infusions of heparinized saline and ceftriaxone (5.25 mg), and s.c. injections of buprenorphine (0.05 mg/kg; first two days) for analgesia. Infusions of methohexital (0.1 mL, 10 mg/mL, i.v.) were administered at the end of NSA acquisition, and after demand assessment to confirm catheter patency. Failure of a patency check resulted in removal of the animal and its data from the study.

### 2.4. Phase 1: SA Acquisition

Acquisition of SA was examined in a total of 68 rats (7–15 for each formulation), using the general apparatus and procedures described previously [46,47,48,49]. Formulations were available during 23-h sessions conducted seven days per week according to an FR 1 schedule, under which each press of an active response lever produced an infusion of either nicotine alone (0.06 mg/kg/inf), EC extract (equivalent nicotine dose), or saline (equivalent volume). This nicotine dose lies on the descending limb of the “inverted-U” SA dose–response curve in which nicotine intake may be limited by its aversive effects. Use of this dose might therefore allow the weaker aversive effects of EC aerosol seen in our prior studies to appear as higher SA compared to nicotine alone. Infusions were delivered in a volume of 0.1 mL/kg in approximately 1 s. Responses on a separate inactive lever were recorded, but had no programmed consequences. Infusions were signaled by offset of the house light and stimulus light over the drug lever throughout the duration of the infusion. After the infusion, the house light was illuminated, but the stimulus light remained off during a subsequent 7-s timeout (TO). During this period, responses on all levers were recorded, but had no programmed consequences. Formulations were available under the FR 1 schedule for at least 10 sessions and until responding stabilized (see below). Criteria for the acquisition of SA were at least 10 infusions per session and a mean active:inactive lever press ratio at least 2:1 for three consecutive sessions. Criteria for the stability of SA were that at least 10 infusions were earned per session, and that there was no trend in the number of infusions per session across three consecutive sessions. Sessions were run seven days per week. Rats typically completed this phase in 17 sessions (range 10–31), by age PD 50 (range 48–62), thereby completing acquisition during late adolescence as commonly defined in rodent studies [50]. 

### 2.5. Phase 2: Elasticity of Demand

Rats that acquired self-administration at FR 1 (6–9 per formulation) were used to assess the elasticity of demand. During this phase, the FR value was increased daily until consumption decreased to zero. The FR values increased according to the sequence: 2, 3, 6, 9, 15, 30 and 60, and these were doubled thereafter. This yielded a progression of unit prices similar to that used in previous studies of EC liquid self-administration [9,12]. Rats typically completed this phase in nine sessions (range 5–18), thereby completing demand assessment in late adolescence to early adulthood [50]. Four rats were included in demand curve analysis, even though their active:inactive ratio did not meet the criterion for acquisition. These rats showed robust increases in active lever responding during FR escalation with no change or a decrease in inactive lever pressing, indicating that nicotine was serving as a reinforcer in these rats. 

### 2.6. Data Analysis

Mean lever presses on the active and inactive levers, the number of infusions, and nicotine intake at each FR value, were the primary dependent measures. For analysis of early acquisition (initial 10 days), mean infusions per session were analyzed via a mixed two-factor analysis of variance (ANOVA) with the formulation as a non-repeated factor and the session as a repeated factor using the Geisser–Greenhouse correction, followed by Tukey post-hoc tests to compare formulations at each session. These data were square-root transformed prior to analyses, in order to correct for non-normality. Mean active and inactive lever presses at stability prior to demand assessment were compared for each formulation by a paired t-test. Inactive lever presses were unusually high (>100) in one rat in the Vuse group (>3.6 SD above the mean), and was detected as an outlier by the Grubbs test (G = 2.8, *p* < 0.01). As such, this rat’s data were excluded from comparison of active and inactive lever pressing. Mean active lever presses and infusions at stability prior to demand assessment were compared between formulations using Brown–Forsyth ANOVA, due to heterogeneity of variance, followed by Dunnett T3 post-hoc tests. Mean infusions during initial acquisition and at stability were pooled across formulations for each sex and compared via an unpaired t-test to examine a main effect of sex. However, sex differences were not examined further, owing to the low number (4–5) of males or females in each of the nicotine control groups. 

To examine the elasticity of demand during the FR escalation phase, demand curve analysis was conducted according to Hursh and Silberberg [32], utilizing the exponential equation

(1) $$logQ=logQ_{0}+k\left(e^{-\alpha Q_{0}C}-1\right)$$

The dependent variable, *Q*, is the quantity consumed. The independent variable, *C*, is the cost of nicotine based on the unit price (FR/unit dose). The free parameters, *Q*_0_ and *α* are estimated from the best-fit function and refer to the maximum level of consumption at zero price (i.e., level or “intensity” of demand) and the rate of decrease in consumption with increases in unit price, respectively. The parameter, *k*, is a constant specifying the range of consumption in log units (set to 2.907 in the present study). The exponential term, *Q*_0_*C*, represents the standardized price of a commodity, correcting for variations in price due to potential differences in the potency of the commodities being compared. The *α* parameter is considered a measure of reinforcing efficacy or “essential value” (i.e., the degree to which a given commodity (e.g., drug) is capable of maintaining behavior under constraints of increasing price). The value of *α* is inversely related to the reinforcing efficacy, so that drugs that produce rapidly declining (elastic) demand curves have higher *α* values and lower reinforcing efficacy than demand curves with slower declining (inelastic) demand curves. Other demand measures of interest included: *Q*_0_, the level or intensity of demand as described above; *O*_max_, the maximal response output; and *P*_max_, the unit price at which maximal response output occurred and demand changes from relatively inelastic to relatively elastic (i.e., the point of unit elasticity).

As mentioned above, the unit price at which zero consumption occurred differed between rats, resulting in missing data for some rats at higher unit prices. Consequently, if rats with missing data are excluded from calculations at high prices, the average of consumption at those prices would be larger than the true average if all rats were included. Therefore, to fit group demand curves, it was assumed that rats would continue to exhibit zero consumption at unit prices above the one at which zero consumption occurred. Therefore, missing data were treated as zero consumption. This approach provides a more accurate portrayal of the true group consumption at high unit prices. However, statistical comparison of demand parameters between groups was done via Brown–Forsyth ANOVA, followed by Dunnett T3 post-hoc tests, to compare the means of parameter estimates derived from curves fit to individual–subject data (see Table 1). In order to include consumption at the highest price in the demand assessment, zeros were replaced with 0.01, since zero is undefined on a log scale (see [9]). This ensured that all demand curves included the full range of consumption. 

## 3. Results

**Figure 1:**
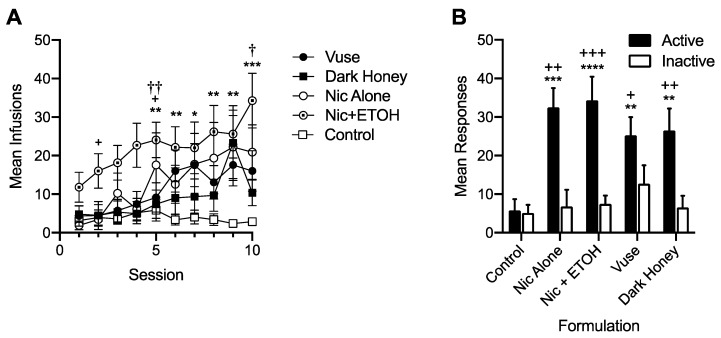
Mean (± the standard error of the mean (SEM)) number of infusions earned per session during the first 10 sessions of the acquisition of self-administration (SA) (panel **A**) and at stability under the FR 1 schedule prior to demand assessment (panel **B**). Each point or bar represents the mean of 9–21 rats. Control is the combined mean of the groups with access to saline alone or saline + ETOH, which did not differ from each other. Because responses during the timeout are not included, the number of active lever responses is equal to the number of infusions for each formulation. Different from Control, * *p* < 0.05, ** *p* < 0.01, *** *p* < 0.001, **** *p* < 0.0001. Different from Dark Honey ^†^ *p* < 0.05, ^††^ *p* < 0.01. Panel A: Different from Vuse ^+^ *p* < 0.05. Panel B: Different from Inactive ^+^ *p* < 0.05, ^++^ *p* < 0.01, ^+++^ *p* < 0.001.

Figure 1 shows the mean number of infusions earned for each formulation during the initial 10 sessions of acquisition (Panel A) and the mean number of active and inactive lever presses for each formulation at stability (Panel B). During initial acquisition, there was a significant main effect of formulation (F = 10.52, *p* < 0.01), session (F = 4.36, *p* < 0.001) and the formulation × session interaction (F = 1.47, *p* < 0.05). Comparisons of the main effect of each formulation showed that all nicotine-containing formulations had higher overall infusion rates compared to the control, and that the Nic+ETOH group had higher overall infusion rates compared to all other nicotine-containing formulations. In addition, Nic+ETOH maintained higher per-session infusion rates than saline from sessions five to ten, whereas those for the other formulations did not significantly differ from control at any session. At stability, all nicotine-containing formulations maintained higher rates of responding on the active lever than the inactive lever, whereas saline formulations did not. Moreover, all nicotine-containing formulations maintained higher active-lever responding and infusions than the control, but they did not differ from each other. There was no sex difference during the first 10 sessions of acquisition. However, females exhibited higher infusion rates for nicotine-containing formulations than males at stability (37.7 ± 4.7 the standard error of the mean (SEM) versus 23.6 ± 3.0 SEM for females versus males, respectively. *t* = 2.53, *p* < 0.05).

**Figure 2:**
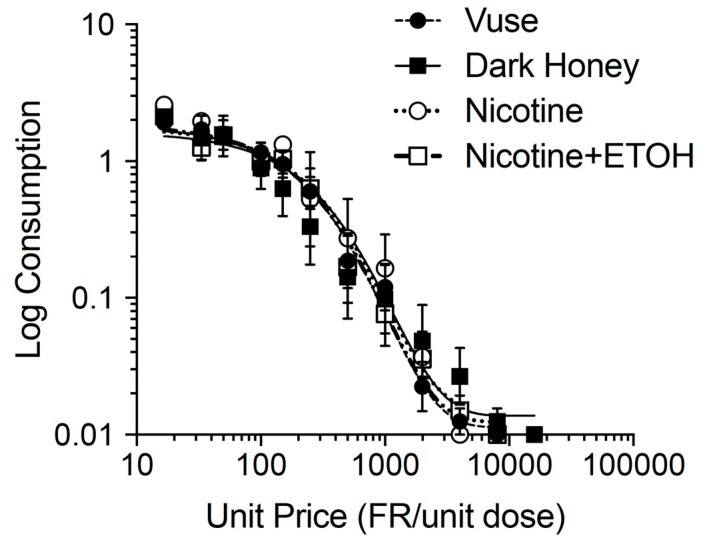
Group demand curves for each formulation in the subsets of rats that acquired self-administration and completed FR escalation (*N* = 6–8 per formulation).

Figure 2 shows group demand curves during the FR-escalation phase of the study. Consistent with the stable response rates at the end of acquisition (Figure 1 panel B), nicotine consumption at the lowest price (i.e., FR 1) did not differ between groups and declined at a similar rate between groups as the unit price increased. Table 1 shows demand curve parameter estimates for individual rats and group means for each formulation. Individual rat and group curves described changes in consumption well, with *r*^2^ > 0.9 for the majority of cases. There were no differences in *α* or any other demand parameter between formulations, indicating no difference in reinforcing efficacy. There was no sex difference in any demand parameter across these formulations.

## 4. Discussion

The main finding of this study is that there was no difference in elasticity of demand (reinforcing efficacy) between nicotine alone and these EC aerosol extracts in adolescent rats. These data suggest that non-nicotine constituents in EC aerosol may not contribute to the reinforcement-related CNS effects of these ECs in adolescents. Our findings have important implications for understanding factors that contribute to EC addiction in youth and any FDA CTP regulatory efforts to reduce it.

The current findings are consistent with our previous studies indicating the similar acquisition and elasticity of demand for nicotine alone and EC liquid in adult rats [9,12], as well as findings from another lab indicating a similar reinforcing efficacy for a different EC aerosol extract and nicotine alone under a progressive ratio schedule of reinforcement in adults [25]. That study also found similar SA of nicotine alone and EC extract under the FR 5 schedule of reinforcement at three of the four nicotine unit doses studied [25]. Overall, these data contrast with some findings indicating greater reinforcing effects of cigarette smoke extract compared to nicotine alone in adults [25,41,51], suggesting that cigarette smoke may have greater abuse liability than EC aerosol. The unique constituent profile of cigarette smoke, which includes higher levels of several behaviorally active non-nicotine constituents than are present in EC liquid or aerosol (e.g., volatile organic compounds such as acetaldehyde) (e.g., [52,53,54]), presumably accounts for this difference. However, one study showed no difference in the reinforcing efficacy between cigarette smoke extract and EC aerosol extract when they were substituted for nicotine alone under a progressive–ratio schedule [25]. Further studies are needed using other models to provide a more comprehensive comparison of the relative abuse liability of cigarette smoke and EC aerosol.

We previously reported attenuated aversive effects of Vuse Menthol EC extract compared to nicotine alone in adolescents in a CTA model [24]. Because nicotine’s aversive effects can limit its abuse liability (e.g., [16,55,56]), this effect could potentially increase the primary reinforcing effects of this extract. While the current data do not support this account, they parallel our prior findings of the similar SA of EC liquids and nicotine alone, despite EC liquids producing attenuated aversive/anhedonic effects in an ICSS model [9,10,12]. There are numerous methodological factors that could account for this discrepancy across models, including nicotine dose, route and contingency. In addition, despite our use of a relatively high nicotine unit dose, nicotine intake in the current SA model may not have been sufficient to allow any aversive effects to manifest. Further evaluation of EC extracts in SA models that exploit nicotine’s aversive effects (e.g., dose escalation models) is needed to examine further the relevance of the reduced aversive effects of EC liquids or extracts seen in ICSS or CTA assays to their reinforcing effects [25]. On the other hand, differences in SA between an EC aerosol extract and nicotine alone have been shown at a very low nicotine dose (0.0075 mg/kg/inf) [25], suggesting further comparison of the nicotine reinforcement threshold (i.e., the lowest reinforcing dose) for EC aerosols versus nicotine alone is warranted. Such work could have important implications for setting nicotine standards for ECs.

Because ETOH was used as a solvent for the preparation of EC extracts, we included controls responding for nicotine or saline combined with the same concentration of ETOH that was in the extracts. ETOH itself was not reinforcing in the SAL + ETOH condition, and did not influence elasticity of demand for nicotine in the nicotine + ETOH condition. Unexpectedly, the nicotine + ETOH group exhibited higher infusion rates during acquisition compared to the other groups. The fact that this apparent nicotine/ETOH interaction was not observed in the EC extract groups suggests that non-nicotine constituents in the extracts may have blocked the effect. This interpretation is questionable, however, as acquisition and stable performance in the nicotine + ETOH group was similar to what we typically observe for nicotine alone and other tobacco product extracts under these conditions [48]. That is, our data may simply reflect lower-than-normal responding in the other groups, rather than an enhancement of nicotine reinforcement in the nicotine + ETOH group, per se. The reasons for the discrepancy in initial response rates between studies is unclear. Further evaluation of the acquisition of SA of nicotine + ETOH cocktails in adolescents, including dose-response testing for both drugs, is needed to better understand these findings.

The higher self-administration rate at stability in females compared to males in the present study is consistent with several other studies of nicotine self-administration in rodents (e.g., [57,58,59]). A limitation of our study is the small sample size for each sex, resulting in inadequate power to examine sex differences within individual groups. However, our goal was not to examine sex differences per se, but to simply model the heterogeneity of participants in human studies. Inclusion of both sexes is also required by NIH if there is no justification for using only one sex. Further research is needed to examine whether the abuse liability of different EC formulations varies by sex.

## 5. Conclusions

This study provides the first characterization of the primary reinforcing effects of EC extracts in adolescent rats. Our findings suggest that non-nicotine constituents may not play a significant role in the reinforcement-related CNS effects of ECs in adolescents, thereby supporting the FDA CTP’s focus on nicotine content for setting product standards to reduce the addictiveness of ECs and other tobacco products. To the extent that similar findings are obtained with new ECs, the present study also suggests that those ECs could be deemed substantially equivalent to current products if their nicotine levels are similar, at least in terms of their reinforcement-related CNS effects. Nonetheless, non-nicotine constituents in these ECs could influence their abuse liability through peripheral sensory effects (e.g., taste, smell), which can contribute to the abuse liability of ECs and other tobacco products (e.g., [60,61]), or could produce addiction-related CNS effects if their levels in ECs were increased. Further research is needed to evaluate whether these conclusions generalize to conditions that model some additional aspects of EC use in humans. For example, the current EC extracts were prepared using a fixed set of puffing parameters, but vaping topography varies considerably across users, and as a function of vaping experience [2,4,62]. Given that differences in puffing topography can influence constituent levels in EC aerosols [2,4,63], EC extracts prepared using other clinically relevant puffing protocols may differ from nicotine alone in this model.

## Figures and Tables

**Table 1:** Individual exponential demand curve parameters across formulations (*k* = 2.907).

Nicotine						Nicotine + ETOH					
ID #	*α*	*Q*_0_	*P*_max_	*O*_max_	*r* ^2^	ID #	*α*	*Q*_0_	*P*_max_	*O*_max_	*r* ^2^
1	0.0010660	4	42.2	56.1	0.6	7	0.0009008	2.16	92.0	66.5	0.79
2	0.0002490	2.51	287.5	240	0.95	8	0.0000860	5.09	415.6	700.0	0.96
3	0.0001998	1.91	471.2	299.1	0.99	9	0.0003445	0.90	581.1	174.0	0.86
4	0.0001455	3.21	384.7	410.7	0.99	10	0.0002182	1.68	493.7	274.7	0.96
5	0.0001593	1.98	570.1	375.1	0.97	11	0.0002273	1.44	551.1	263.6	0.96
6	0.0002260	1.48	536.5	264.4	0.97	12	0.0001247	1.43	1012	481.0	0.98
						13	0.0007798	2.30	100.9	76.8	0.98
						14	0.0008966	3.11	64.7	66.8	0.89
						15	0.0002182	1.68	491.7	273.9	0.96
Mean	0.00034093	2.52	382	274.2	0.91	Mean	0.0004218	2.20	422.5	264.1	0.93
SEM	0.00014589	0.38	79.9	51.1	0.06	SEM	0.0001138	0.42	101.4	70.6	0.02
**Vuse**						**Dark Honey**					
16	0.0003346	2.12	254.1	178.6	0.96	24	0.0010910	4.72	35.0	54.8	0.96
17	0.0003082	1.69	345.2	193.9	0.93	25	0.0005600	1.24	259.6	106.7	0.93
18	0.0001185	1.6	949.6	504.3	0.97	26	0.0000490	2.54	1444.9	1220.6	0.93
19	0.0002000	2.03	443.4	298.8	0.98	27	0.0002127	1.40	602.2	281.0	0.94
20	0.0003380	0.85	626.2	176.8	0.84	28	0.0010740	0.36	458.8	55.6	0.75
21	0.0005376	1.38	243.3	111.2	0.95	29	0.0000830	2.12	1024.6	720.2	0.97
22	0.0006735	3.14	85.1	88.7	0.78	30	0.0000792	2.54	893.4	754.2	0.95
23	0.0002529	2.54	280.5	236.3	0.95	31	0.0011260	3.60	44.4	53.1	0.73
Mean	0.0003454	1.92	367.5	204.4	0.92	Mean	0.0005344	2.32	540.4	368.5	0.9
SEM	0.0000637	0.25	97.7	47.9	0.02	SEM	0.0001743	0.49	175.0	151.0	0.03
